# Editorial: Insights in systems endocrinology: 2021

**DOI:** 10.3389/fendo.2023.1223931

**Published:** 2023-07-13

**Authors:** M. Seldin, D. Stefanovski

**Affiliations:** ^1^ The Department of Biological Chemistry, University of California, Irvine, Irvine, CA, United States; ^2^ Department of Clinical Studies - New Bolton Center, School of Veterinary Medicine, University of Pennsylvania, Philadelphia, PA, United States

**Keywords:** systems biology, big data, machine learning, mathematical modeling, systems endocrinology

Systems endocrinology is a rapidly growing field that leverages novel methodologies such as systems biology, big data, and machine learning to broaden our understanding of the complex interaction of hormones and their action on regulating normal physiological function as well as the etiology of complex diseases such as diabetes, obesity, heart disease, and many others. With this Research Topic of Systems Endocrinology, we attempted to illustrate the breadth of the field. Starting from the top level (populations of individuals) and ending on the molecular level, this Research Topic highlights the work of four international groups.

Starting from the most extensive system level (multiple cohorts of subjects), the article by Zhou et al. presents the systematic review and meta-analysis of studies dealing with the effects of SGLT2i in comparison to other antihyperglycemic drugs and their impact on hepatic fibrosis and steatosis. This study highlighted the diversity of drug responsiveness, where liver outcomes differed in categories such as patient age. Then, moving down to the level of a single endocrine system, Morettini et al. present a novel model of the effect of amino acids on insulin kinetics. Here, the authors implemented a simple model to capture glucose tolerance test dynamics in response to amino acid changes, where AA-mediated increases in insulin secretion were not predicted to be significantly impacted by T2D status. Expanding on models of metabolic homeostasis, Shi et al. performed a pathway-informed analysis of potential resveratrol target genes which were relevant for impacting preeclampsia. By integrating several datasets using gene ontology, keg, PPI and molecular docking approaches, several key driver pathways were elucidated, such as RAGE and HIF1a. Finally, to capture the complexity of inter-organ signaling in the context of large-scale datasets, Bankier and Michoel present an elegant review detailing usage of eQTLs in guiding hormone signaling and tissue coordination.

In sum, these studies reflect the diversity of approaches and methods to utilize systems-based approaches in investigation of endocrinology.

## Author contributions

All authors listed have made a substantial, direct, and intellectual contribution to the work and approved it for publication.

